# More than one way to bind to cholesterol: atypical variants of membrane-binding domain of perfringolysin O selected by ribosome display†

**DOI:** 10.1039/d0ra06976k

**Published:** 2020-10-21

**Authors:** Aleksandra Šakanović, Nace Kranjc, Neža Omersa, Marjetka Podobnik, Gregor Anderluh

**Affiliations:** Department of Molecular Biology and Nanobiotechnology, National Institute of Chemistry Ljubljana Slovenia gregor.anderluh@ki.si; Biosciences Doctoral Program, Biotechnical Faculty, University of Ljubljana Ljubljana Slovenia

## Abstract

Herein, we report a high-throughput approach for the selection of peripheral protein domains that bind specifically to cholesterol in lipid membranes. We discovered variants of perfringolysin O, with non-conserved amino acid substitutions at regions crucial for cholesterol recognition, demonstrating an unprecedented amino acid sequence variability with binding ability for cholesterol. The developed approach provides an effective platform for a comprehensive study of protein lipid interactions.

## Abstract 2

By using developed ribosomal display, we discovered variants of perfringolysin O, a pore forming toxin from bacteria *Clostridium perfringens*, with non-conserved amino acid substitutions at regions crucial for cholesterol recognition.

## Graphical abstract

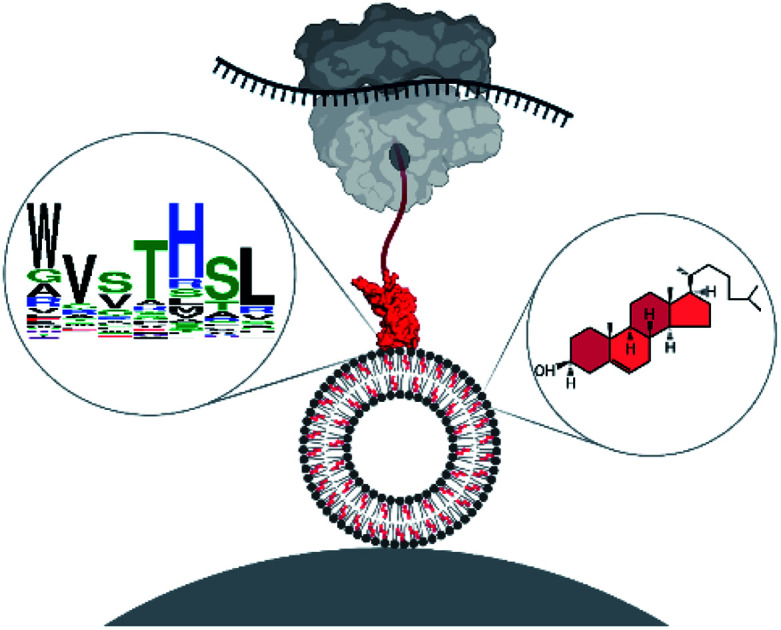

Specific lipid recognition and the subsequent recruitment of peripheral proteins to particular cellular sites enable crucial biological processes and represent core molecular mechanisms that enable attack and defense by cellular and pathogen-associated proteins.^1–3^ Moreover, in chemical biology, lipid targeting by small protein domains is needed for targeted localization of proteins to subcellular compartments or model lipid vesicles and it represents a widely used approach to study the distribution and metabolism of various cellular lipids.^4–8^ Despite significant advances in recent years, lipid membrane-related applications and membrane-binding proteins with engineered properties, such as specific lipid selectivity, are scarce.^9^

High throughput approaches of directed protein evolution can be used to investigate functional consequences of mutations from a large pool of protein variants, thus overcoming the bottleneck of classical mutagenesis. These approaches have been successfully applied to study, improve, or modify the binding properties of soluble proteins or peptides,^10^ to increase the catalytic functions of enzymes,^11^ to improve the activity of pore-forming proteins and to increase the expression and detergent-stability of integral membrane proteins.^12–14^ The directed protein evolution strategies have not yet been applied to explore the large and complex landscape of peripheral protein-lipid interactions.

Here, we developed a ribosome display approach (Scheme 1) to select peripheral membrane proteins, using domain 4 (D4) of perfringolysin O (PFO), a cholesterol-dependent cytolysin (CDC) from bacterium *Clostridium perfringens*. PFO monomer has an elongated shape consisting of four domains (Fig. S1a, ESI†), of which D4 ensures specific binding to cholesterol-containing lipid membranes. The critical structural elements responsible for the membrane binding and cholesterol-dependent activity of CDCs have been substantially explored.^15–21^ They include undecapeptide loop (458-ECTGLAWEWWR-468 in PFO) and a conserved threonine–leucine pair (T490 and L491 in PFO) in loop 1, which assures cholesterol specificity by a yet unknown mechanism (Fig. S1b, ESI†).

**Scheme 1:**
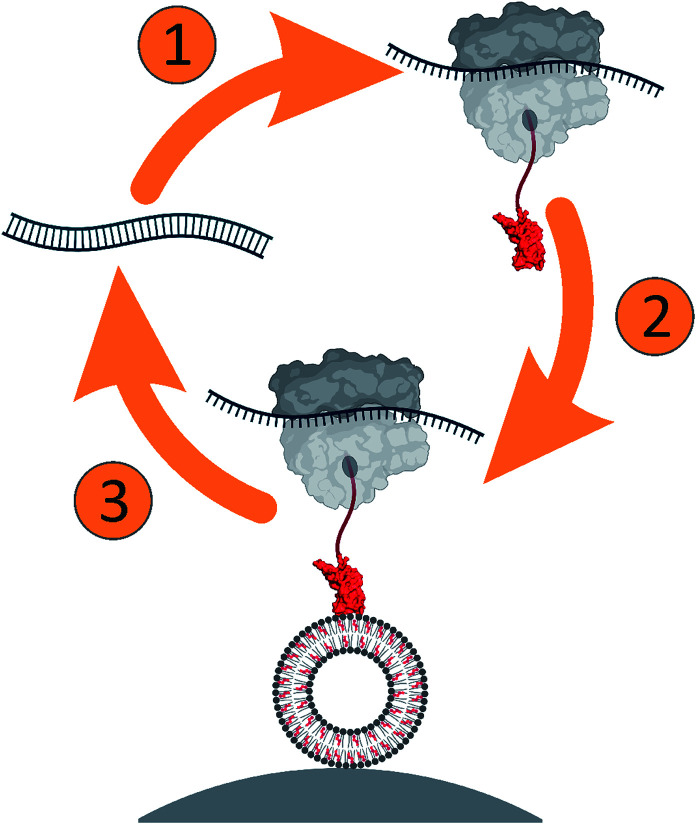
Ribosome display was developed to select PFO variants for selective binding of cholesterol in lipid membranes. The approach is composed of (1) *in vitro* transcription and translation of the DNA library, (2) selection of the protein–ribosome–mRNA complex against lipid vesicles, and (3) amplification of the obtained RNAs for the next round of selection.

To investigate the contributions of the amino acid residues in the D4 loops of PFO to membrane binding, we created the D4 gene library consisting of seven degenerated codons in the NNK scheme (where N is any nucleotide and K is G or T) for the following residues: A401, V403, E458, T460, R468, T490, and L491 (Fig. S1b and c, ESI; † throughout the paper, the identity of a particular variant is indicated by the amino acids at these positions, whereas wild-type D4 is designated as AVETRTL). The designed D4 gene pool comprised a theoretical diversity of 3.4 × 10^10^. Residues at the sites that were randomized are largely conserved in CDCs and are likely to be involved in initial contact with the target membrane due to their position and solution-exposed side chain orientation (Fig. S1b, ESI†).^22–25^ This scaffold, therefore, allows comprehensive exploration of the functional consequences of amino acid substitutions in the D4-binding region.

The D4 gene library was subjected to *in vitro* transcription/translation and affinity selection against cholesterol-containing small unilamellar vesicles (SUVs) immobilized on streptavidin-coated magnetic beads (Scheme 1). We performed thorough characterization of the selection assay during the development (Fig. S2 and S3, ESI†). To obtain deep insights into the variability of amino acids at randomized positions, we used next generation DNA sequencing (NGS) and analyzed the sequences of the input library and libraries after the second and fourth selection round.^26,27^ The analysis of all obtained sequences clearly shows the expected very high number of mismatches at randomized positions and the negligible number of mismatches at other positions, at most 3.5% per codon (Fig. S3a, ESI†). Mismatches at positions other than the randomized are likely to occur due to mutations during PCR amplification and DNA template preparation. However, mismatches, aside from the randomized positions, were far from the loops involved in membrane binding. As such, they are unlikely to play a role during initial binding and were thus not further assessed.

We noticed an increased number of sequences with higher occurrence in the library after affinity screening in comparison to a high number of sequences with low occurrence in the input library (Fig. S3b, ESI†).

This clearly indicates successful selection and enrichment of specific D4 variants. To understand the residue preferences at each variable position, sequence logos were generated from sequences that covered all seven randomized positions and were present in two or more copies (Fig. 1). Population of protein variants present after affinity screening demonstrated an enrichment of amino acids chemically similar to those of the wild-type. In particular, after four selection rounds, clear enrichment of T490, or a very similar serine, and L491 can be observed, representing a cholesterol recognition motif.^25^ Accordingly, the most common amino acids after affinity selection at the second and fourth position are the same as those present in the wild-type, namely the valine and threonine. Furthermore, tryptophan, which is known to have a high affinity for the interfacial region of membrane-binding proteins,^1,28^ is significantly enriched at the first randomized position instead of alanine with a small hydrophobic side chain.

**Fig. 1:**
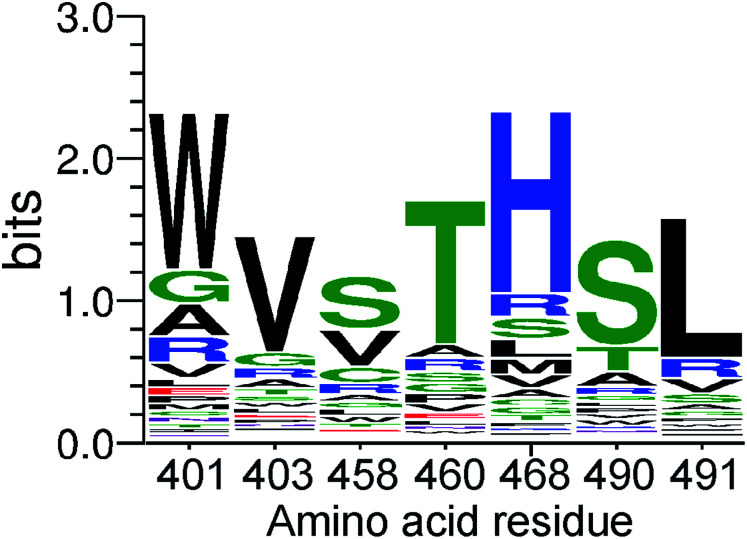
A sequence logo depicting the site specific amino acid preferences of the PFO variants at each randomized position after four rounds of selection.

Moreover, positively charged histidine predominates at position 468, instead of bulky arginine. Conversely, the most significant difference between the wild-type amino acid sequence and the residues from the affinity-selected population of protein variants with the highest enrichment was observed at the third randomized position. Short polar and non-polar or positively charged side chain of arginine were enriched at position 458 instead of the original glutamate with a negatively charged side chain (Fig. 1). This position can clearly tolerate substitutions without abolishing the binding ability to cholesterol-containing lipid vesicles.

We further analyzed the enrichment of individual sequence variants. The top three variants were WVSTHSL, WVVTHSL, and WVCTHSL (bold in Fig. S3d, ESI†). These variants differ only in the residue at the third randomized position. In addition, when we examined shorter reads with the identified amino acids WV at the first two randomized positions and other or unknown amino acids at the remaining five positions as well as reads with THSL at the last four positions (which were also among the most abundant variants), the overall enrichment exceeded a frequency of several percent (Fig. S3d, ESI†). Thus, enrichment indeed resulted in the selection of highly similar, wild-type-like sequences.

Overall, the sequencing results indicate that the majority of amino acids in the most common D4 variants not only remain conserved or similar to the wild-type, but also that PFO residues at the membrane interface can tolerate substitutions without significantly abolishing their binding ability to cholesterol-containing lipid vesicles. Moreover, our results indicate that the proposed cholesterol recognition motif can tolerate certain changes that imply a previously undetected plasticity of the otherwise conserved D4 region of CDCs. For example, a highly unusual WVVTHVW variant was retrieved that differs significantly from the proposed cholesterol recognition motif T490–L491; however, its enrichment was significantly lower compared to that of most abundant variants. The described amino acid preferences at positions explored by extensive mutagenesis and identification of rare variants with biochemically diverse residues at evolutionary conserved positions cannot be detected by conventional site-directed mutagenesis, which consists of an analysis of only a few of the most radical mutations. This demonstrates the efficiency of high-throughput approaches, such as ribosome display, used in this study.

To experimentally validate the results of the affinity-based selection by the ribosome display approach, we expressed and purified the full-length PFO, variant WVVTHSL, which was among the most abundant (Fig. S3d, ESI†) and the WVVTHVW variant that showed significant differences in the proposed cholesterol recognition motif. Their activity and binding properties were compared with those of the wild-type PFO. First, we assayed the ability of the selected variants to provoke lysis of red blood cells (Fig. 2a).

**Fig. 2:**
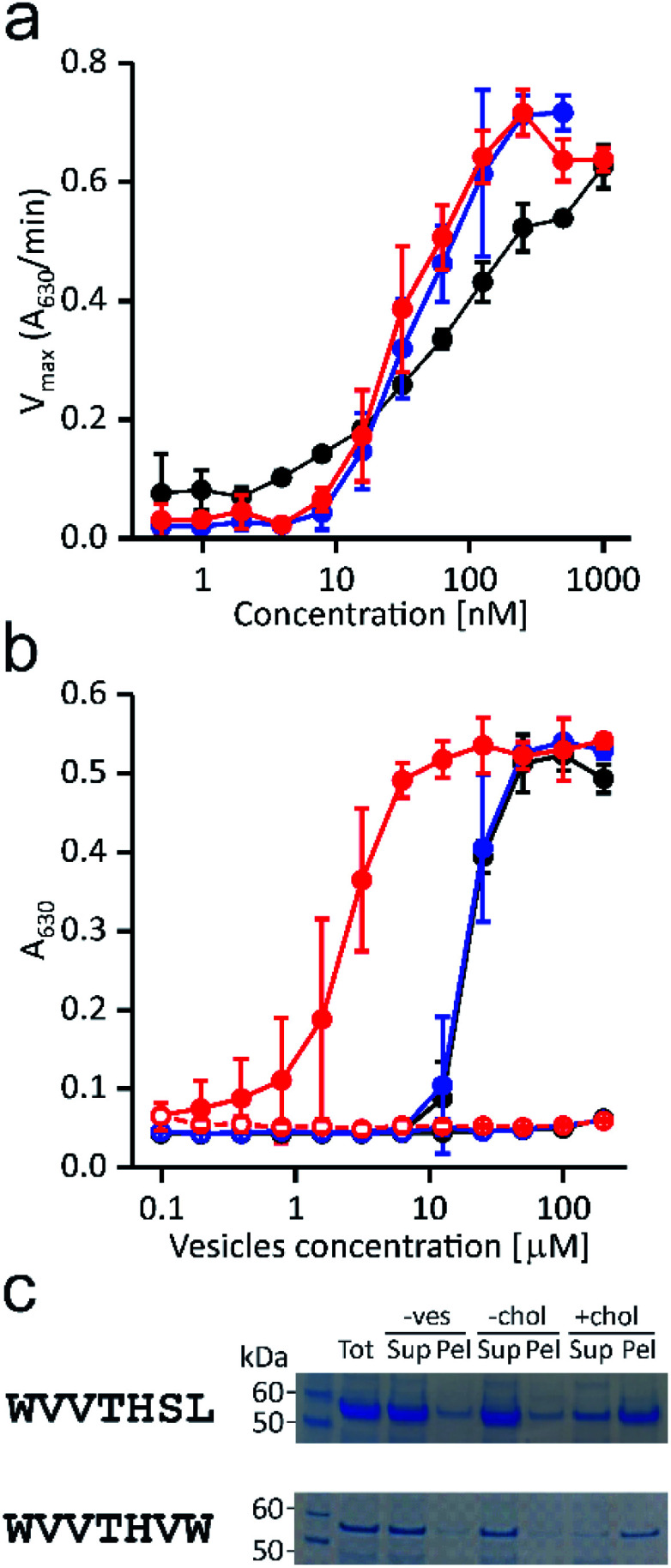
Functional properties of the selected variants. (a) Hemolysis of bovine red blood cells induced by the wild-type perfringolysin O (black), WVVTHSL (blue), and WVVTHVW variants (red); *n* = 3, mean ± S.D. (b) Inhibition of hemolytic activity induced by pre-incubation of 200 nM proteins with different concentrations of lipid vesicles composed of POPC : chol (solid circles) or POPC (open circles). Colors are as in (a); *n* = 3, mean ± S.D. (c) WVVTHSL and WVVTHVW variants binding to POPC (−chol) or POPC : cholesterol 1 : 1 (mol/mol) (+chol) MLVs detected on SDS PAGE gel after vesicle sedimentation. Tot, total amount of protein applied; Sup, supernatant; Pel, pellet; −ves, the control experiment without vesicles. The data for the wild-type PFO are shown on Fig. S2b, ESI.†

Despite profound differences in the otherwise conserved T490–L491 motif, the WVVTHVW variant proved to be hemolytic, as the calculated concentration required to reach half of the maximal activity rate (36 ± 2 nM) was lower than in wild-type PFO (81 ± 20 nM). In addition, slightly better hemolytic activity of the WVVTHSL variant was also observed, as the dose required to cause 50% hemolysis (47 ± 21 nM) was lower compared to the wild-type PFO.

Binding of the PFO variants to cholesterol-containing lipid vesicles was demonstrated by two independent assays. In the hemolytic inhibition assay, serial two-fold dilutions of vesicles were pre-incubated with fixed amounts of the protein, and subsequently exposed to bovine erythrocytes. The hemolytic activity of the tested variants dramatically decreased after pre-incubation with the cholesterol-containing vesicles, indicating stable binding of the proteins to the vesicles (Fig. 2b). Moreover, inhibition of the hemolytic activity of the WVVTHVW variant occurred at lower vesicle concentrations than those required to inhibit the same amount of wild-type or WVVTHSL variant.

The observed higher affinity for the vesicles of the WVVTHVW variant could be a consequence of the predominant hydrophobic substitutions, which could preferably mediate initial membrane attachment by hydrophobic interactions. Conversely, lipid vesicles without cholesterol, *i.e.*, composed solely of 1-palmitoyl-2-oleoyl-*sn-glycero*-3-phosphocholine (POPC), did not affect hemolytic activity (Fig. 2b). Consistent with the pre-sequestration assay, the cholesterol-specific binding of protein variants was also confirmed by a sedimentation assay. Binding of protein variants to cholesterol-containing multilamellar vesicles (MLVs) was clearly observed, whereas no protein binding to MLVs consisting of only POPC was detected (Fig. 2c).

To independently confirm whether selected variants exhibit cholesterol specificity typical for PFO and CDCs, we employed the enzyme-linked immunosorbent assay (ELISA), where the wild-type PFO and both variants bound specifically to nanomolar concentrations of cholesterol, and none of them bound to POPC nor to the cholesterol analog cholesteryl-acetate (Fig. 3) (3β-hydroxy-5-cholestene 3-acetate), which represents a negative control, as it is very similar to cholesterol yet CDCs do not bind to it due to its changed 3β-hydroxyl group.^17,18^

**Fig. 3:**
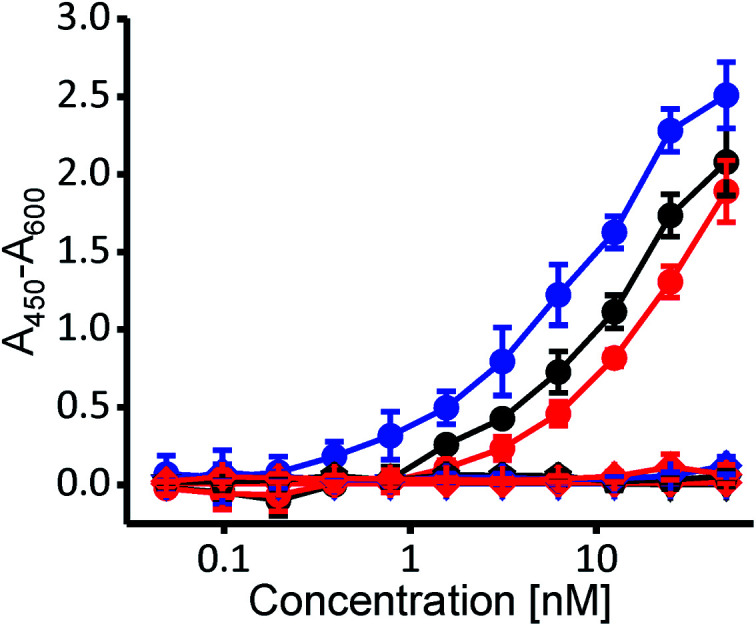
ELISA measurement of binding of the wild type PFO (black), WVVTHSL (blue) and WVVTHVW (red) to cholesterol (solid circles), POPC (triangle) and cholesteryl-acetate (diamond). *n* = 3, mean ± S.D.

Altogether, these results are consistent with the ribosome display affinity selection using SUVs and clearly show that the two selected protein variants exhibit cholesterol-dependent binding that is similar to that of the wild-type PFO. Most importantly, as with the wild-type PFO, both variants remain hemolytically active.

In summary, we have developed a modified ribosome display approach that can be used for affinity selection of peripheral membrane proteins with lipid-specific targeting. To develop the approach and to discover new variants with changed properties, we used a well-established cholesterol-binding protein domain previously employed as a probe to monitor cholesterol distribution in cellular membranes.^29–34^ Surprisingly, we were able to detect D4 sequences that were substantially different from naturally occurring ones but with a retained capability to bind to cholesterol-containing membranes. The D4 sequence span for cholesterol binding is clearly broader than anticipated, which is greatly advantageous for synthetic biology and development of engineered lipid binding domains for other applications. Our results also clearly demonstrate the potential of ribosome display as a selection method for other protein domains or lipids.^35,36^

## Conflicts of interest

There are no conflicts of interest to declare.

## Supplementary Material

RA-010-D0RA06976K-s001
