# Supplementary material for: More than one way to bind to cholesterol: atypical variants of membrane-binding domain of perfringolysin O selected by ribosome display
Source: RSC Adv. 2020 Oct 21;10(63):38678–82. doi: 10.1039/d0ra06976k (PMC9057304; doi:10.1039/d0ra06976k)
Supplement: RA-010-D0RA06976K-s001 [file RA-010-D0RA06976K-s001.pdf]

Electronic Supplementary Material (ESI) for RSC Advances.
This journal is © The Royal Society of Chemistry 2020

ELECTRONIC SUPPLEMENTARY INFORMATION

More than one way to bind to cholesterol: Novel variants of membrane-binding domain of perfringolysin O selected by ribosome display

Aleksandra Šakanović,[a,b] Nace Kranjc,[a,c] Neža Omersa,[a] Marjetka Podobnik,[a] and Gregor Anderluh *[a]

a Department of Molecular Biology and Nanobiotechnology, National Institute of Chemistry, Ljubljana, Slovenia, E-mail: gregor.anderluh@ki.si

b Biosciences Doctoral Program, Biotechnical Faculty, University of Ljubljana, Ljubljana, Slovenia.

c Present address: Department of Life Sciences, Imperial Colleage London, South Kensington Campus, London, United Kingdom.

Table of Contents

Materials and Methods 2

Supplementary results 7

Supplementary figures 9

Supplementary references 12

Materials and Methods

1.1 Materials. The plasmid pRDV and *E. coli* strain MRE600 were provided by dr. Plückthun's lab[1], and the D4 gene library was obtained from Eurofins Genomics (Germany). All primers were purchased from Integrated DNA Technologies (USA), restriction enzymes were from New England Biolabs (USA), RNase inhibitor RiboLock, NTPs, T4 DNA ligase, T7 RNA polymerase, and DNAse I were from ThermoFisher Scientific (USA). 1-palmitoyl-2-oleoyl-*sn*-glycero-3-phosphocholine (POPC), cholesterol (Chol), 1-oleoyl-2-(12-biotinyl (aminododecanoyl))-*sn*-glycero-3-phosphoethanolamine (Biotin-PE) were from Avanti Polar Lipids (Alabaster, USA). Lissamine rhodamine B 1,2-dihexadecanoyl-*sn*-glycero-3-phosphoethanolamine (rhodamine DHPE) was from Invitrogen (USA). All other chemicals were from Sigma (USA), unless stated otherwise.

1.2 Preparation of ribosome display DNA template and affinity selection. The DNA template for ribosome display was prepared by PCR in two steps as described previously.[2] In short, the D4 gene library starting with S386 (PDB ID: 1PFO) (Fig S1c), which contained random nucleotide triplets at selected positions in the NNK scheme (where N is any nucleotide and K is G or T), was constructed from the synthetic DNA. The D4 gene library, wild-type D4, and D4 mutant LTRTRLT for control affinity selection (all without stop codons) were first amplified with library-specific primers (sense primer D4_forII: CAAAGGATCCACAGAGTATTCTAAGG and antisense D4_revIII: CCATATAAAGCTTATTGTAAGTAATACTAGATCC) that introduced the *Bam*HI and *Hin*dIII restriction sites underlined in the sequence of the primer. The resulting PCR product was purified by agarose gel electrophoresis and digested by *Bam*HI and *Hin*dIII restriction enzymes. The digested and purified D4 library was ligated into the *Bam*HI- and *Hin*dIII-digested pRDV. Subsequently, the ligation mixture was amplified using the sense primer specific to the region upstream of the T7 promoter (T7B2: CGAAATTAATACGACTCACTATAGGGAGACCACAACGG) and the antisense primer specific to the region downstream of the tolA sequence (TolA: AGAAAGAAAGCGGCAACTGAAGCTGCTGAAAAAGCCAAAGCAGAA), both encoded by pRDV vector. The resulting PCR product encoding for T7 promoter, the sequences for stabilizing 3' and 5' RNA's stem loops, D4 library member and a tolA spacer sequence, was purified by agarose gel electrophoresis and as such used for coupled *in vitro* transcription and translation reactions in an *E. coli* S30 system.

E. coli S30 ribosomes were purified from the MRE 600 strain (ATCC 29417) as described previously.[3] The optimal conditions for *in vitro* transcription and translation reactions, in particular with regard to optimal concentrations of $Mg(OAc)_2$, KOAc, and trehalose for each batch of *E. coli* S30, were determined using the β-lactamase assay with nitrocefin. The transcription and translation reactions were carried out for 35 min at 37 °C with shaking at 700 rpm and in 22 µl of reaction mixture containing the following components: 50 mM Tris HOAc at pH 7.4, 27 mM NH_4OAc, 1 mM of each amino acid, 1.2 mM ATP, 0.87 mM GTP, CTP, and UTP, 0.6 mM cAMP, 0.2 mg/ml *E. coli* tRNA, 4.5% trehalose, 2.3 mM DTT, 34 µg/mL folinic acid, 80 mM $LiKAcPO_4$, 12.3 mM $Mg(OAc)_2$, 210 mM KOAc, 3.6 µM anti-ssrA oligonucleotide, 0.9 µL T7 RNA polymerase, 11 µL *E. coli* S-30, RiboLock RNAse inhibitor, and 80 ng template DNA. The translation reaction was stopped after 35 min by cooling on ice for 2 min and five-fold dilution with ice-cold washing

buffer (50 mM Tris-acetate, pH 7.5, 150 mM NaCl, 50 mM magnesium acetate, 0.1% bovine serum albumin (BSA), with added 2.5 mg/mL heparin). To eliminate any insoluble components, the diluted reaction mixture was centrifuged at 13 000 × *g* for 5 min at 4 °C.

Streptavidin magnetic beads (Dynabeads M-280, ThermoFisher Scientific) for vesicle immobilization (20 µL/sample) were first pre-washed twice with the same volume of solution A (100 mM NaOH and 50 mM NaCl) and then equilibrated by washing three times with 100 µL washing buffer (50 mM Tris-acetate, pH 7.5, 150 mM NaCl, 50 mM magnesium acetate, and 0.1% BSA). After removal of the washing buffer, 20 µL of small unilamellar vesicles (SUVs) composed of 1 mM POPC, 1 mM Chol, and 0.5 mol % Biotin-PE were added to prepared beads and gently shaken at 4 °C for 1 h. The optimal conditions for SUVs binding and maximal capacity of the beads to bind SUVs were determined by preparing POPC:Chol SUVs, with added 0.5 mol % of Biotin-PE and 0.5% rhodamine DHPE and measuring the fluorescence in magnet pelleted beads and in supernatant. Unbound vesicles were removed by three washes with 100 µL of washing buffer. To prevent non-specific binding, the bound vesicles were treated with 0.5% BSA in washing buffer at 4 °C overnight. Bound vesicles were washed three times with ice-cold washing buffer. Next, the supernatant from the centrifuged transcription and translation mixture was added and incubated for 1 h at 4 °C with gentle shaking. The selection pressure was performed by successive washing steps with 100 µL of the ice-cold washing buffer as follows: 3 × 2 min for the first and second round, 2 × 2 min and 2 × 3 min for the third round, and finally 2 × 2 min, 2 × 3 min, and 2 × 5 min for the fourth round. After each selection round, the retained protein-mRNA-ribosome complexes were dissociated with the elution buffer (50 mM Tris-acetate, pH 7.5, 150 mM NaCl, 25 EDTA, and 50 µg/mL *Saccharomyces cerevisiae* RNA) and rigorous shaking for 10 min. The RNA from the eluted fraction was purified using High Pure RNA Isolation Kit (Roche Diagnostics, Switzerland), and immediately after isolation, it was reversely transcribed by the High Capacity cDNA RT Kit (Life Technologies, USA) and amplified using library-specific primers. Amplified DNA was used to prepare the DNA template for the next selection round or, after the second and fourth round, for NGS sequencing library preparation.

In order to control the preparation of the DNA template and the progress of the affinity selection, Sanger sequencing was used. Individual clones were prepared by transformation of the *E. coli* DH5α strain with the D4 library ligated to the pRDV vector. The plasmids from 50 clones from the input library as well as almost 100 clones from the libraries after the second and fourth round of selection were isolated using the Monarch Plasmid MiniPrep Kit (New England Biolabs, USA) according to manufacturer's instructions and sequenced using the T7 primer and Sanger sequencing. The sequences were analyzed with Vector NTI software (ThermoFisher Scientific, USA).

1.3 NGS library preparation and sequencing. The input D4 gene library and libraries after the second and fourth round of affinity selection were amplified using D4-specific primers (sense primer D4_forII: CAAAGGATCCACAGAGTATTCTAAGG and antisense D4_revIII: CCATATAAAGCTTATTGTAAGTAATACTAGATCC). Agarose gel electrophoresis was used to purify 350 bp long PCR products, which were subjected to NGS library preparation using the Ion Plus Fragment Library Kit (ThermoFisher Scientific, USA) with 100 ng of the DNA template. Adapter and barcode ligation, size selection, nick repair, and five amplification cycles using Platinum PCR SuperMix and NGS library-specific primers were carried out according to the manufacturer's instructions described in the Ion Torrent protocol. The quality and concentration of the prepared NGS libraries were analyzed with the Agilent 2100 Bioanalyzer and the corresponding

High Sensitivity DNA kit (Agilent Technologies, USA). All three NGS libraries were diluted to a concentration of 15 pM, mixed in equimolar ratio, and subjected to emulsion PCR and enrichment steps using the Ion OneTouchTM 2 System and Ion PGMTM Hi-QTM OT2 kit. The Ion SphereTM Quality Control kit and the Qubit 3.0 fluorimeter were used to determine the quality of the prepared NGS library according to the manufacturer's instructions. The prepared NGS library was sequenced with the Ion 314 Chip. Signal processing, base calling, and adapter sequence trimming were performed using Torrent Suite software. The final quality-filtered numbers of reads that were used in the analysis were 33 497, 18 261, and 16 403 for the input library and libraries after the second and fourth round, respectively.

1.4 Analysis of high-throughput sequencing data and variant identification. Sequencing reads were aligned to the nucleotide sequence of the PFO D4 domain using LAST.[4] Mismatches, insertions, and deletions were detected and counted in each alignment to determine the error rate and mutation frequency at each position in the nucleotide sequence. Alignments were further processed to account for any insertion or deletion artefacts arising from PCR amplification or sequencing and to enable successful *in silico* translation to the amino acid sequence. The amino acid frequency was calculated for each randomized position of the D4 domain library. The relative change in the amino acid frequency at each randomized position was calculated by dividing the amino acid frequency from each sampled selection round with the amino acid frequency of the D4 library before the selection. Data processing, calculations, and visualization were performed using the Python packages NumPy, SciPy, Pandas, and Seaborn. Sequence logos were generated with WebLogo 3.[5]

1.5 Preparation of multilamellar and unilamellar lipid vesicles. To prepare multilamellar vesicles (MLVs), lipids were dissolved in chloroform to 10 mM solutions and mixed in the desired ratio. After evaporation of the solvent, the lipid film was hydrated with a suitable buffer depending on its further use. For vesicles used in affinity selection, the lipid film was hydrated in washing buffer (50 mM Tris acetate, pH 7.5, 150 mM NaCl, 50 mM magnesium acetate, 0.1% BSA), while the lipid film was hydrated in 50 mM Tris acetate, pH 7.5 and 150 mM NaCl for vesicles used in the hemolysis inhibition assay or sedimentation assay. To prepare small unilamellar vesicles (SUVs), MLVs were subjected to six freeze-thaw cycles followed by sonication with an ultrasonic probe on ice for 25 min in 10 s pulses with 35% amplitude. For the preparation of vesicles used in the hemolysis inhibition assay, MLVs were extruded 21 times through polycarbonate filters with 800 nm pore size using a two-syringe extruder (NanoSizerTM Extruder, T&T Scientific Corporation, USA). The size distribution profile of the vesicles was routinely checked by dynamic light scattering (Zetasizer Nano-ZS, Malvern Instruments, UK).

1.6 Plasmid preparation and protein purification. The gene coding for wild-type perfringolysin O (PFO) was subjected to mutagenesis, which led to a silent mutation and the formation of the *Bst*BI restriction site at the beginning of the coding region for the D4 domain. The *Xho*I restriction site, the DNA sequence encoding N-terminal 6 × His-tag, followed by the recognition site for the TEV protease and the *Avr*II restriction site at the 5' end and the *Mlu*I restriction site at the 3' end, was introduced by PCR primers, and the resulting wild-type PFO gene fragment was cloned into the pET8c vector using the *Xho*I and *Mlu*I sites. The prepared construct was named pHT-PFO wt. PCR amplification of the gene fragments coding for D4 LTRTRLT, D4 WVVTHSL, and D4 WVVTHVW, identified with control Sanger sequencing, was used to introduce the *Bst*BI and *Mlu*I restriction sites at the 5' and 3' ends of the D4 gene fragments, respectively. Amplified D4 frag-

ments were extracted from agarose gel using the Monarch Gel Extraction kit (New England Biolabs, USA) and cloned into pHT-PFO wt using the *Bst*BI and *Mlu*I restriction sites. The resulting constructs were named pHT-PFO LTRTRLT, pHT-PFO WVVTHSL, and pHT-PFO WVVTHVW. Recombinant proteins were expressed and purified as described previously.[6] Briefly, freshly transformed *E. coli* BL21(DE3) pLysS cells in LB medium supplemented with 100 μg/mL ampicillin and 30 μg/mL chloramphenicol were grown at 37 °C. When the culture reached $OD_{600\ nm}$ ~ 0.5, 0.5 mM isopropyl β-D-1-thiogalactopyranoside was added to induce protein production. After 20 h of cultivation at 20 °C with 180 rpm shaking, cells were harvested by centrifugation at 4000 × *g* for 15 min at 4 °C.

For protein purification, cells were resuspended in lysis buffer (50 mM NaH_2PO_4, 300 mM NaCl, and 10 mM imidazole, pH 8.0) at a ratio of 10 mL/g wet bacterial mass, lysed by sonication and, after removal of the cell debris by centrifugation and supernatant filtration, subjected to nickel affinity chromatography (Ni-NTA Superflow, Qiagen) as described previously.[6] Purified proteins were dialyzed against Tris-HCl buffer (50 mM Tris-HCl, pH 7.4, and 150 mM NaCl) using Slide-a-Lyzer with MWCO 10 kDa (ThermoFisher Scientific, USA); protein purity was confirmed by SDS-PAGE and Coomassie staining. Aliquots of the purified proteins were flash frozen in liquid nitrogen and stored at −20 °C until use.

1.7 Hemolytic activity of purified variants. The hemolytic activity of PFO variants was measured as described previously.[7] In short, bovine erythrocytes stored at 4 °C in Alsever's preservative were washed with erythrocyte buffer (20 mM Tris-HCl, pH 7. 4, and 140 mM NaCl), and 3-5 resuspension and centrifugation steps at 800 × *g* for 4 min were performed. Subsequently, the sedimented erythrocytes were diluted in erythrocyte buffer to an absorbance of ~1 at 630 nm (A_{630}). The erythrocyte suspension (100 μL) was added to two-fold serial dilutions of tested proteins (100 μL) in 96-well clear microtiter plates. The extent of lysis was quantified by direct measurement of the absorbance at 630 nm every 20 s for 20 min at 25 °C using the Synergy MX microplate reader (Biotek, USA).

1.8 Hemolysis inhibition assay. Proteins were pre-incubated with lipid vesicles composed of POPC or POPC:Chol 1:1 (mol:mol) and then the hemolytic activity of the protein-lipid vesicle mixtures against bovine erythrocytes was measured. A two-fold serial dilution of lipid vesicles (200 μM–0.01 μM) extruded through a polycarbonate filter with a pore size of 800 nm was prepared, and 50 μL of each lipid vesicle concentration was mixed with the same volume of 200 nM proteins. After 15 min incubation at 22-24 °C, 100 μL of erythrocyte suspension diluted to an absorbance of ~1 at 630 nm was added to the vesicle-protein mixture. The hemolytic activity was determined by measuring the absorbance at 630 nm immediately after the addition of erythrocytes.

1.9 Vesicle sedimentation assay. Proteins (2 μM) were incubated with a 2000 × molar excess of MLVs in a buffered system (50 mM Tris-acetate and 150 mM NaCl, pH 7.4) for 30 min at 22-24 °C. The mixtures were then centrifuged at 16 000 × *g* for 30 min at 4 °C. The supernatants were transferred to fresh microtubes, while unbound or loosely bound protein molecules were removed from the pellets by resuspension in 100 μL buffer solution and centrifugation under the same conditions. Afterwards, unbound proteins in the supernatant and bound proteins in the pellet were subjected to SDS-PAGE followed by protein detection using SimplyBlue SafeStain (Thermo Fisher Scientific, USA).

1.10 ELISA assessment of protein variant binding to various lipids. Lipids (POPC, cholesteryl-acetate, and Chol) dissolved in chloroform to 2 mM were diluted in ethanol to 20 µM, and 100 µL of this prepared lipid solution was used to coat the wells of the microtiter plate. After overnight solvent evaporation at 22-24 °C, 200 µL of 3% BSA in Tris-buffered saline buffer (TBS, 10 mM Tris-HCl, pH 7.4, and 150 mM NaCl) was added to each well and incubated for 2 h at 22-24 °C, followed by three washes with 200 µL TBS. Next, two-fold serial dilutions of proteins from a concentration of 100 nM were prepared in TBS with 1% BSA, and 100 µL was incubated in lipid-coated microtiter wells for 1 h at 22-24 °C. Subsequently, unbound protein molecules were removed by six washes with 200 µL of TBS. Bound proteins were detected with monoclonal mouse Penta-His antibodies (Qiagen, Germany) diluted at a ratio of 1:1000 in TBS supplemented with 1% BSA, and 100 µL was added to each well. After 1 h incubation and six washes with 200 µL TBS, 100 µL of anti-mouse antibodies conjugated with horseradish peroxidase diluted at a ratio of 1:5000 was added to each well and incubated for 1 h. Unbound antibodies were rinsed by six washes, and detection of bound complexes was performed with 3,3′,5,5′-tetramethylbenzidine substrate according to manufacturer's instructions. The intensity of the color developed after addition of horseradish peroxidase-substrate was measured at 490 nm with a reference of 630 nm with the Synergy MX microplate reader (Biotek, USA).

Supplementary results

2.1 Characterization of the D4 selection assay

The D4 starting from S386 (PDB ID: 1PFO) can be expressed and purified as an independent domain with ability to bind in the cholesterol- specific manner.[8] Therefore, the D4 gene library (Fig. S1c) from S386 was used as a template for the *in vitro* transcription/translation reaction in the conditions which enables the formation of stable complexes between ribosome, mRNA and protein. Ternary complexes were affinity selected against cholesterol-containing small unilamellar vesicles (SUVs) immobilized on streptavidin-coated magnetic beads (Scheme 1). The maximum binding capacity and the complete coverage of the magnetic beads with the target SUVs were determined by measuring fluorescence in the supernatant and pelleted beads after binding of SUVs, which were composed of 1-palmitoyl-2-oleoyl-*sn*-glycero-3-phosphocholine (POPC) and cholesterol (Chol) as well as fluorescently labeled phosphatidylethanolamine. By using dynamic light scattering, we confirmed that adding ternary protein-ribosome-mRNA complexes did not damage lipid vesicles (data not shown). Each selection round consisted of proteins in the protein-ribosome-mRNA complexes binding to magnetic bead-immobilized POPC:Chol SUVs, washing away unbound complexes, subsequent isolation and reverse transcription of the RNA from the bound fraction, and PCR amplification to produce the DNA template for the next round of selection (Scheme 1).

We first characterized clones after the selection process with Sanger sequencing, which was used to provide initial insight into the affinity selection and to cover the whole length of the gene for D4. We indeed observed an enrichment of particular clones, as we detected eleven copies of the WVVTHSL variant among hundred analyzed sequences after four rounds of selection. One variant, VGKMAFA, was present in three copies, and four variants were present in two copies (WVVTHVW, RGARGMG, LVVTHSL, and LTRTRLT). All other variants were present in only one copy. This result indicates that numerous unique sequences were likely present in the sample that were not affinity-selected but rather represent a background noise, which is not uncommon in ribosome display and other affinity-based methods for *in vitro* evolution approaches.[9-11] Apart from the variants that bind with high affinity to cholesterol-containing membranes, we also expected other variants in our experimental system; namely, those that bind with low affinity due to changes in positions important for binding as well as sequences that result in hydrophobic D4 surfaces, which could non-specifically associate with bead-immobilized SUVs or other components of the assay, thereby further contributing to the background. The binding of DNA to liposomes is another possible source of the background noise.[11] Indeed, about 97% of identified sequences after four rounds of selection occurred only once and their corresponding frequency in sequenced library was 0.029%. Thus, validating the membrane-binding properties of selected variants is of paramount importance, particularly for those that are present in low numbers of copies.

Therefore, we tested whether one of these observed variants, LTRTRLT, was either affinity-purified or just a component of the background noise. This variant is interesting as it exhibits the inversion of amino acids T490-L491 in the proposed cholesterol recognition motif and additional substitutions of the studied amino acids. However, we did not expect it to significantly associate with lipid membranes. We mixed equimolar amounts of wild-type AVETRTL and LTRTRLT DNA and determined whether the LTRTRLT variant can compete with the wild-type for association with lipid vesicles during ribosome display. Sanger sequencing of 24 clones after a separate experiment with four selection rounds revealed that the mutant variant LTRTRLT was not present,

indicating successful affinity selection of the wild-type D4. We further validated this variant by preparing a recombinant full-length LTRTRLT. Functional analysis of its activity revealed that it was not hemolytically active (at concentrations up to 1 μM), in contrast to the wild-type PFO (Fig. S2a). We also confirmed that LTRTRLT does not bind to lipid membranes by using the sedimentation assay, in which proteins were added to multilamellar vesicles (MLVs), and the bound fraction was detected on SDS-PAGE after pelleting by centrifugation. As expected, the wild-type PFO bound considerably to vesicles composed of POPC and Chol at a ratio of 1:1 (mol:mol), but not to POPC vesicles. In contrast, the LTRTRLT mutant did not exhibit such binding specificity and did not associate with cholesterol-containing vesicles (Fig. S2b). Thus, LTRTRLT, which exhibited no or very low affinity towards cholesterol-containing SUVs and was not detected after affinity selection in control ribosome display experiment, was treated as a negative control. The established ribosome display conditions were used in all subsequent experiments.

Supplementary figures

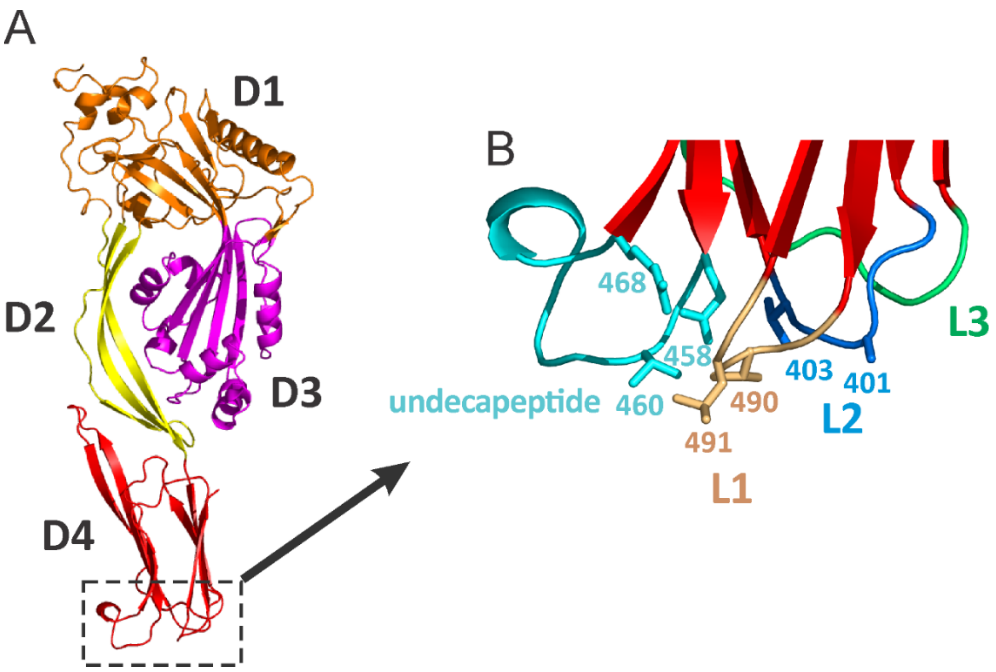

C

TCC	ACA	GAG	TAT	TCT	AAG	GGA	AAA	ATA	AAC	TTA	GAT	CAT	AGT	GGA
S	T	E	Y	S	K	G	K	I	N	L	D	H	S	G
**NNK**	TAT	**NNK**	GCA	CAG	TTT	GAA	GTA	GCC	TGG	GAT	GAA	GTT	TCA	TAT
**X**	Y	**X**	A	Q	F	E	V	A	W	D	E	V	S	Y
GAC	AAA	GAA	GGA	AAT	GAA	GTT	TTA	ACT	CAT	AAA	ACA	TGG	GAT	GGA
D	K	E	G	N	E	V	L	T	H	K	T	W	D	G
AAT	TAT	CAA	GAT	AAA	ACA	GCT	CAC	TAT	TCA	ACA	GTA	ATA	CCT	CTT
N	Y	Q	D	K	T	A	H	Y	S	T	V	I	P	L
GAA	GCT	AAT	GCA	AGA	AAT	ATA	AGA	ATA	AAA	GCA	AGA	**NNK**	TGT	**NNK**
E	A	N	A	R	N	I	R	I	K	A	R	**X**	C	**X**
GGC	CTT	GCT	TGG	GAA	TGG	TGG	**NNK**	GAT	GTT	ATA	AGT	GAA	TAT	GAT
G	L	A	W	E	W	W	**X**	D	V	I	S	E	Y	D
GTT	CCA	TTA	ACA	AAT	AAT	ATA	AAT	GTT	TCA	ATA	TGG	GGA	ACA	**NNK**
V	P	L	T	N	N	I	N	V	S	I	W	G	T	**X**
**NNK**	TAC	CCT	GGA	TCT	AGT	ATT	ACT	TAC	AAT					
**X**	Y	P	G	S	S	I	T	Y	N					

Fig. S1 The crystal structure of the Perfringolysin O with enlarged loops of the D4 domain and its nucleotide and amino acid sequence with denoted randomized positions relevant for this study. (A) The structure of perfringolysin O (PDB ID: 1PFO) with four domains, colored and labeled. (B) Enlargement of the loops at the membrane binding site of D4. The three loops (L1–L3) and the undecapeptide motif are labeled with different colors. The side chains of residues that were randomized in the library are shown as sticks and denoted by numbers. (C) The nucleotide and amino acid sequence of the D4 domain as cloned into the pRDV vector to prepare the D4 DNA library; NNK (bold) indicates random nucleotides.

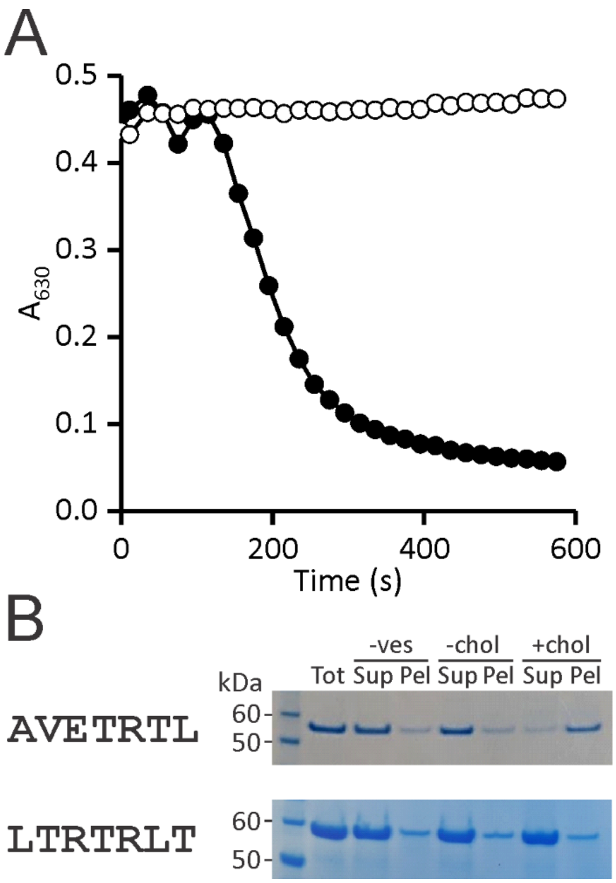

Fig. S2 Hemolytic activity and binding of the negative control mutant LTRTRLT. (A) Hemolytic activity of 3.1 nM wild-type perfringolysin O (AVETRTL; solid circles) and 900 nM LTRTRLT mutant (open circles). Bovine red blood cells were resuspended in 20 mM Tris-HCl, pH 7. 4, and 140 mM NaCl. (B) The binding of 2 µM proteins to MLVs composed of POPC (-chol) or POPC:Chol (+chol) as detected by the vesicle sedimentation assay on SDS-PAGE. The control experiment did not contain MLVs (-ves). Tot, total amount of protein applied; Sup, supernatant; Pel, pellet.

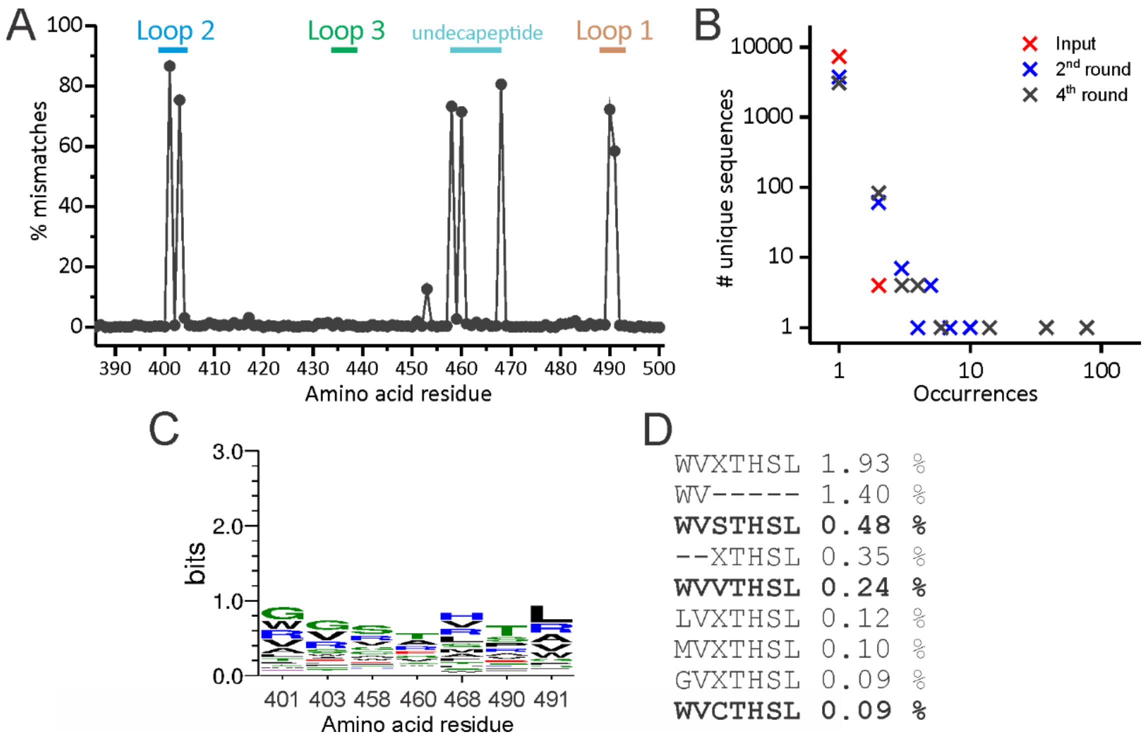

Fig. S3 The characterization of sequence reads obtained with ribosome display. (A) The observed mismatches after four selection rounds. The loops in D4 are indicated and colored as in Figure 1. (B) The abundance of unique sequences in the initial library and after two and four rounds of selection. (C) Sequence logos composed of sequences that cover all seven randomized positions and were represented in at least two copies after two (184 sequences) rounds of selection. The amino acids are colored according to the chemical properties of their side chains (black, hydrophobic; green, polar; blue, positively charged; red, negatively charged). (D) The most frequent sequence variants obtained after four rounds of selection. Sequences that cover all seven positions are indicated in bold. The most frequent sequence variants obtained from shorter reads are also presented. X denotes any amino acid residue, and a dash represents missing sequence data.

Supplementary references

1. H. K. Binz, P. Amstutz, A. Kohl, M. T. Stumpp, C. Briand, P. Forrer, M. G. Grutter and A. Pluckthun, *Nature Biotechnology*, 2004, **22**, 575-582.
2. C. Zahnd, P. Amstutz and A. Pluckthun, *Nature Methods*, 2007, **4**, 269-279.
3. B. Dreier and A. Pluckthun, *Methods Mol Biol*, 2011, **687**, 283-306.
4. S. M. Kielbasa, R. Wan, K. Sato, P. Horton and M. C. Frith, *Genome Research*, 2011, **21**, 487-493.
5. G. E. Crooks, G. Hon, J. M. Chandonia and S. E. Brenner, *Genome Research*, 2004, **14**, 1188-1190.
6. E. Lasič, M. Lisjak, A. Horvat, M. Božič, A. Šakanović, G. Anderluh, A. Verkhratsky, N. Vardjan, J. Jorgačevski, M. Stenovec and R. Zorec, *Scientific reports*, 2019, **9**, 10957.
7. M. Kisovec, S. Rezelj, P. Knap, M. M. Cajnko, S. Caserman, A. Flašker, N. Žnidaršič, M. Repič, J. Mavri, Y. Ruan, S. Scheuring, M. Podobnik and G. Anderluh, *Scientific Reports*, 2017, **7**, 42231.
8. Y. Shimada, M. Nakamura, Y. Naito, K. Nomura and Y. Ohno-Iwashita, *Journal of Biological Chemistry*, 1999, **274**, 18536-18542.
9. T. A. Whitehead, A. Chevalier, Y. Song, C. Dreyfus, S. J. Fleishman, C. De Mattos, C. A. Myers, H. Kamisetty, P. Blair, I. A. Wilson and D. Baker, *Nature Biotechnology*, 2012, **30**, 543-548.
10. N. C. Wu, G. Grande, H. L. Turner, A. B. Ward, J. Xie, R. A. Lerner and I. A. Wilson, *Nature Communications*, 2017, **8**, 15371.
11. T. Nishikawa, T. Sunami, T. Matsuura, N. Ichihashi and T. Yomo, *Anal Chem*, 2012, **84**, 5017-5024.
